# TPP1 is associated with risk of advanced precursors and cervical cancer survival

**DOI:** 10.1371/journal.pone.0298118

**Published:** 2024-05-09

**Authors:** Qiao-Li Wang, Caifeng Gong, Xiang-Yu Meng, Min Fu, Hui Yang, Fuxiang Zhou, Qiuji Wu, Yunfeng Zhou

**Affiliations:** 1 Department of Radiation and Medical Oncology, Hubei Key Laboratory of Tumor Biological Behaviors, Zhongnan Hospital of Wuhan University, Hubei Cancer Clinical Study Center, Wuhan, Hubei, China; 2 Department of Medical Oncology, Dana-Farber Cancer Institute, Boston, MA, United States of America; 3 Department of Medical Oncology, National Cancer Center/National Clinical Research Center for Cancer/Cancer Hospital, Chinese Academy of Medical Sciences and Peking Union Medical College, Beijing, China; 4 Health Science Center, Hubei Minzu University, Enshi, China; 5 Department of Oncology, Tongji Hospital, Tongji Medical College, Huazhong University of Science and Technology, Wuhan, China; Istituto Nazionale Tumori IRCCS Fondazione Pascale, ITALY

## Abstract

It is unclear how telomere-binding protein TPP1 interacts with human telomerase reverse transcriptase (hTERT) and influences cervical cancer development and progression. This study included all eligible 156 cervical cancers diagnosed during 2003–2008 and followed up through 2014, 102 cervical intraepithelial neoplasia (CIN) patients, and 16 participants with normal cervix identified at the same period. Correlation of expression of TPP1 and hTERT in these lesions was assessed using Kappa statistics. TPP1 was knocked down by siRNA in three cervical cancer cell lines. We assessed mRNA expression using quantitative real-time polymerase chain reaction and protein expression using tissue microarray-based immunohistochemical staining. We further analyzed the impact of TPP1 expression on the overall survival of cervical cancer patients by calculating the hazard ratio (HR) with 95% confidence intervals (CIs) using the multivariable-adjusted Cox regression model. Compared to the normal cervix, high TPP1expression was significantly associated with CIN 3 and cervical cancers (*P*<0.001 for both). Expressions of TPP1 and hTERT were highly correlated in CIN 3 (Kappa statistics = 0.50, *P* = 0.005), squamous cell carcinoma (Kappa statistics = 0.22, *P* = 0.011), and adenocarcinoma/adenosquamous carcinoma (Kappa statistics = 0.77, *P* = 0.001). Mechanistically, knockdown of *TPP1* inhibited the expression of hTERT in both mRNA and protein levels. High expression of TPP1 (HR = 2.61, 95% CI 1.23–5.51) and co-high expression of TPP1 and hTERT (HR = 2.38, 95% CI 1.28–4.43) were independently associated with worse survival in cervical cancer patients. TPP1 and hTERT expression was correlated and high expression of TPP1 was associated with high risk of CIN 3 and cervical cancer and could predict a worse survival in cervical cancer.

## Introduction

In 2020, the Global Cancer Observatory estimated 604,127 new cervical cancer cases and 341,831 cervical cancer-related deaths and cervical cancer ranked as the fourth most common and deadly cancer among women [1]. Increasing young women aged 15 to 49 years have been diagnosed with cervical cancer during the last decade, especially in low- and middle-income countries [2,3]. Early detection of precancerous lesions and early-stage cervical cancer could substantially improve patient survival [4]. Despite great efforts in identifying novel biomarkers for cervical cancer in recent years, valid biomarkers are still limited.

Telomere-binding protein TPP1 (TINT1-PTOP-PIP1) is a vital component of the telomere-binding protein shelterin complex, protecting chromosome ends from cellular DNA damage [5]. TPP1 initiates DNA repair machinery by recruiting telomerase to telomeres, stimulating telomerase activity, and promoting telomere elongation in telomerase-positive cells [6,7]. It also combines with the protection of telomeres 1 (POT1) to form a stable heterodimer, thereby maintaining genomic stability and regulating telomerase-mediated telomere extension [7,8]. All these functions suggest that TPP1 plays a vital role in preventing carcinogenesis by maintaining telomere length and activity. Human telomerase reverse transcriptase (hTERT) is associated with cell stemness, cell proliferation, and resistance to chemotherapy and radiotherapy in various cancers [9,10]. TPP1 may regulate telomere length partly through hTERT [11–13]. Inhibition and silent mutation of either hTERT or TPP1 activity could shorten the telomere length and hinder the telomere functions in human cells [14]. But whether TPP1 could serve as an independent biomarker in pathogenesis and prognosis in cervical cancer has not been fully investigated.

In this retrospective study, TPP1 expression was assessed in a continuum of cervical lesions, from normal cervix, cervical intraepithelial neoplasia (CIN) 1 to 3, to cervical cancer in an institutional patient cohort. We analyzed the association of TPP1 expression with cervical cancer pathogenesis and prognosis. We further explored the role of TPP1 in affecting hTERT expression. This study may provide important clues of using TPP1 as both a risk biomarker and a prognostic factor in cervical cancer.

## Materials and methods

### Study design

A total of 274 cervical tissue samples were collected in this study. All eligible cervical cancer patients (n = 156) were identified in Zhongnan Hospital of Wuhan University (Wuhan, China) between January 1, 2003, and December 31, 2008. Another 102 cervical intraepithelial neoplasia (CIN) tissues and 16 normal cervical tissue samples were collected from Fanpu Biotech Inc. (Guilin, Guangxi, China) during the same study period. Cervical cancer patients were aged > 18 years, diagnosed with cervical cancer as primary cancer during the study period and had complete medical records, and received radical hysterectomy, with or without chemo- or radiotherapy. CIN patients received either cervical biopsy or surgical resection. Normal cervical tissues were collected from participants who had hysteromyoma resection but without other cervical diseases. Cervical cancer patients were followed up through outpatient visits in the hospital and a regular phone call by the Clinical Follow-up Unit. Follow-up started from the date of cancer diagnosis to death from any cause, loss to follow-up, or end of the study (January 31, 2014). This study was approved by the Institutional Ethical Review Board of the Zhongnan Hospital of Wuhan University (ethical approval number: 2014054). Written informed consent was obtained from participating patients for the use of their tissue samples.

Clinical characteristics of cervical cancer cases were collected from medical records, which included age, human papillomavirus (HPV) infection status, tumor diameter, International Federation of Gynecology and Obstetrics (FIGO) stage, differentiation, lymphatic invasion, vaginal invasion, para-uterus invasion, and pathological subtype. Overall survival (OS) time was defined as the date of surgery to the date of death from any cause or the last date of follow-up, whichever came first.

### Tissue microarray-based immunohistochemical (IHC) staining

Tissue microarrays (TMA) containing cervical cancer, CINs, and normal cervix tissues were constructed. Hematoxylin and eosin staining was used to confirm the primary pathological diagnosis by an institutional pathologist. For each specimen, three or four 1.0-mm diameter sections were circled and implemented for TMA construction [15]. Immunohistochemistry was performed to assess TPP1 and hTERT expression following the manufacturer’s instructions. Primary antibodies against TPP1 (Abcam, ab57595, dilution of 1:100) and hTERT (Abcam, ab183105, dilution of 1:30) were incubated on 4 μm-section samples for 1.5 hours at room temperature. TPP1 was expressed in the nucleus thus low expression of TPP1 was defined as <50% of positive cells with TPP1 staining, and high expression was defined as ≥50% of positive cells with TPP1 staining. We categorized hTERT expression levels according to a previously published method [16], which was rated by a combination of intensity of staining (negative, weak, moderate, or strong) and percentage of positive cells (<10%, 10–50%, 50–75%, or >75%). For each cervical tissue sample, two blocks per case were evaluated, and an average score was applied. Two pathologists blinded to the outcome independently assessed the protein expression manually using a predefined rating system shown in **S1 Table**. Any discrepancy in scores between the two pathologists was solved by re-evaluating and rescoring the samples together.

### RNA extraction and reverse transcription, and protein extraction

Total cell RNA was extracted using TRIzol reagent (Invitrogen, US). The concentration and quality of RNA were evaluated by a Nanodrop 2000 Spectrophotometer (Thermo Fisher Scientific). Reverse transcribed cDNA was synthesized from 0.1 μg-5 μg of total RNA by the Recert AidTM first strand cDNA Synthesis Kit (Fermentas, Canada) at 42°C for 60 min, followed by 70°C for 5 min and 4°C afterward. For the protein extraction, cells were digested using 1 ml trypsin-EDTA solution (Beyotime, China) and then lysed with 500 μL RIPA lysis buffer per 100 packed cell volumes (PCVs).

### Cell transfection, quantitative real-time polymerase chain reaction (qPCR) and Western blot

The plasmids (purchased from GenePharma, Shanghai) were used to knock down *TPP1* with a TPP1-siRNA sequence of 5’-GTGGTACCAGCATAGCCT-3’ in three human cervical cancer cell lines, HeLa, C33a, and SiHa, which were purchased from the Institute of Life Sciences, Chinese Academy of Science (Shanghai, China). Cells were plated in two six-well plates at 2 × 105 cells per well. After 24 hours of incubation, 5 μL of transfection reagent TurboFect (Thermo Fisher Scientific, USA) was mixed with 8 μL of siRNA in 500 μL DMEM, and then the mixture was added to the cell culture medium. Cells were collected 48 hours after transfection to perform transfection validation and RNA and protein extraction. Transfection efficiency assessed by the immunofluorescence method considered both intensity and percentage of fluorescent cells in the images. TPP1-siRNA cell lines were compared with the FAM-marked negative control (siNC group) and blank control (mock group).

Real-time qPCR was performed with SYBR Premix Ex Taq (Takara, Japan) in a 12.5 μL reaction volume, plus 0.5 μL PCR forward primer (10 μM) and 0.5 μL reverse primer (10 μL), 0.5 μL ROX Reference Dye II 50 μM (Thermo Fisher Scientific, USA), 2 μL DNA template, and 9 μL ddH_2_O, using the CFX Connect Real-Time qPCR detection system (Bio-Rad, US). PCR condition was set as 94°C for 5mins followed by 94°C for 30s, 60°C for 30s, and 72°C for 60s. A total of 40 cycles were applied. The melting curve and amplicon size were checked. Primers designed for *GAPDH*, *hTERT*, and *TPP1* were shown in **S2 Table**. All values were normalized to *GAPDH*, and the 2-DDCt method was used to estimate the fold change of gene expression over control samples.

Western blot was carried out to evaluate TPP1 and hTERT protein expression. Primary antibodies targeting TPP1 (ab57595, Abcam, USA, dilution of 1:750), hTERT (ab32020, Abcam, USA, dilution of 1:1000), and β-actin (Santa Cruz, US, dilution of 1:2000) were incubated at 4°C overnight. ECL (Advansta, USA) was used to visualize the specific bands. Auto radiographs were recorded onto X-ray films (Eastman Kodak Co, USA), and ImageJ software was applied to analyze the signal intensity of bands.

### Gene expression of TPP1 in publicly available databases

An eligible microarray dataset (GEO accession number: GSE7803), including the gene expression of normal cervical tissues, CINs, and cervical cancer tissues, was used to assess the expression of *TPP1* in different cervical lesions by ANOVA test. The Oncomine gene expression array dataset was queried to analyze *TPP1* mRNA expression in cervical cancer and normal tissues. The defined parameters used to filter datasets were *P*-value < 1E−4 (Student’s t-test), fold-change > 2, and genes ranking in the top 10%. One dataset from Zhai Cervix met the criteria and was selected to assess the expression of *TPP1* in cervical cancer tissues and normal tissues.

### Statistical analysis

The association of TPP1 expression and clinical-pathological characteristics was assessed using the chi-square test. The agreement of TPP1 and hTERT expression levels in different cervical lesions was assessed by bivariate correlation analysis (Cohen’s Kappa statistics) [17]. To assess the association between high TPP1 expression and different cervical lesions, odds ratios with 95% confidence interval (CI) were estimated using binary logistic regression. The results of *in vitro* analysis are given as the mean ± standard error of the mean (SEM) with a minimum of three repetitions, and Student’s t-test was conducted. Kaplan–Meier curves with a log-rank test were performed to compare the overall survival of cancer patients with different expression levels of TPP1 or together with hTERT. To test the prognostic value of high TPP1 expression or co-high expression of TPP1/hTERT, hazard ratio (HR) with a 95% CI was estimated using multivariate analysis by the Cox proportional hazards model, adjusting for other well-known prognostic factors, i.e., age, HPV infection, tumor diameter, FIGO stage, differentiation, lymphatic invasion, vaginal invasion, para-uterus invasion, pathological subtype, and chemo- or radiotherapy. Statistical analyses were performed in SPSS 26 (SPSS Inc., Cary, NC, USA). All statistical analyses were two-sided, and a *P*-value of < 0.050 was considered statistically significant.

## Results

### TPP1 expression, cervical cancer pathogenesis, and clinical-pathological factors

**Fig 1:**
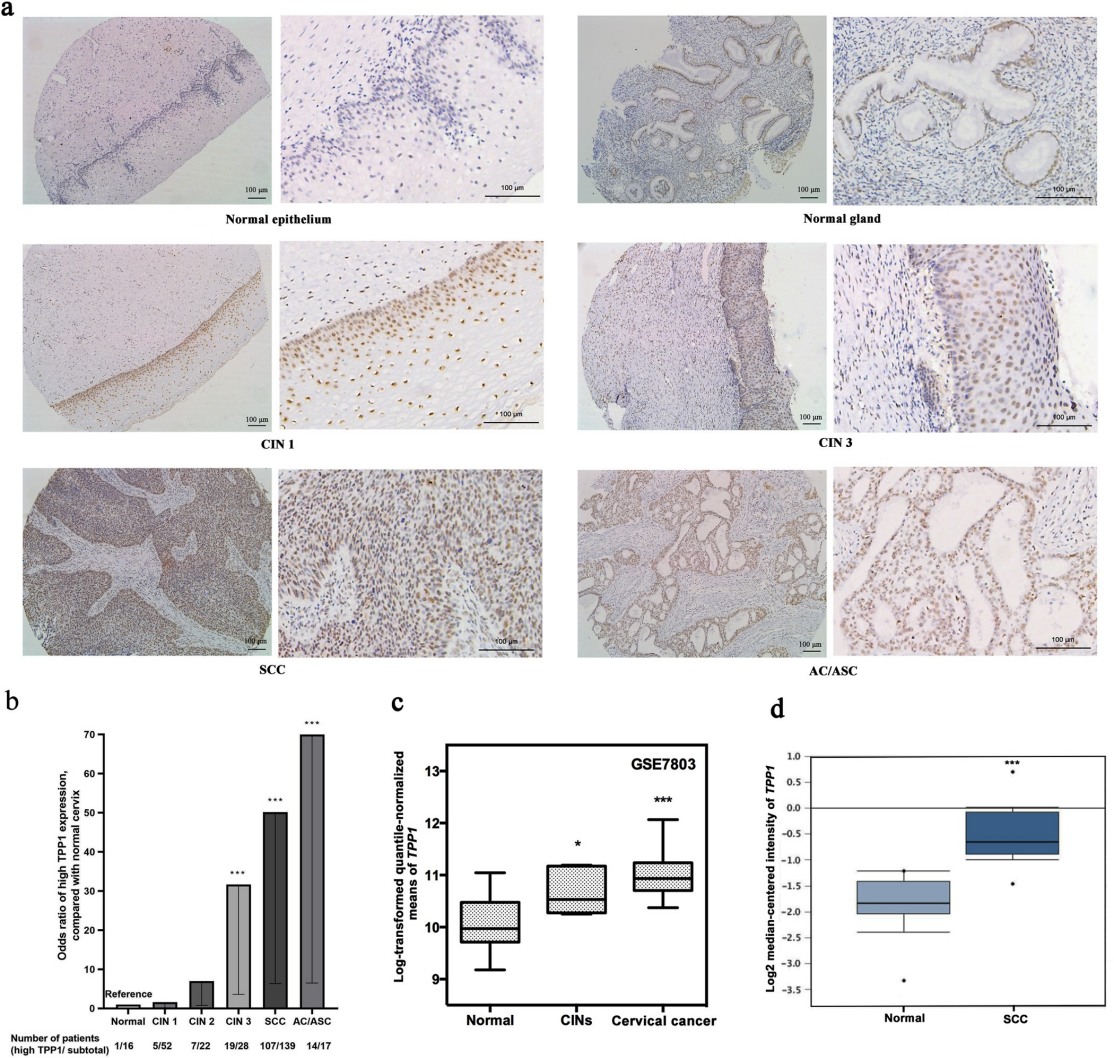
Expression of TPP1 in different cervical lesions. **(a)** Representative results of TPP1 expression in normal, CIN 1, CIN 3, and cervical carcinoma tissues by immunohistochemistry (n = 274, scale: 100 μm). **(b)** Odds ratio of high TPP1 protein expression among CIN and cervical carcinoma tissues compared with the normal cervix (n = 274). **(c)** Gene expression of *TPP1* in the GSE7803 dataset, comparing CIN and cervical cancer with the normal cervix. **(d)** Gene expression of *TPP1* in the Zhai Cervix dataset from Oncomine, comparing cervical squamous cell carcinoma with normal cervix squamous epithelium. Abbreviations: AC: Adenocarcinoma; ASC: Adenosquamous carcinoma; CINs: Intraepithelial neoplasia; SCC: Squamous cell carcinoma; * *P* < 0.05, *** *P* < 0.001.

A total of 16 normal cervices, 52 CIN 1, 22 CIN 2, 28 CIN 3, 139 cervical squamous cell carcinoma (SCC), and 13 cervical adenocarcinomas (AC)/adenosquamous carcinoma (ASC), were eligible for analysis **(Table 1)**. The staining pattern of TPP1 was found to be negative in normal epithelial cells and very weak in normal grands, while it initiated moderate expression in basal cells in CIN1 and extended to suprabasal cells in CIN3, with diffuse strong expression throughout the SCC and AC/ASC lesions **(Fig 1A)**. High expression of TPP1 was detected in 6.3% of normal cervix, 9.6% of CIN 1, 31.8% of CIN 2, 67.9% of CIN 3, 77.0% of SCC and 82.4% of AC/ ASC **(Fig 1B)**. Compared with the normal cervix, a significantly increased proportion of high TPP1 expression was found for CIN 3 (*P* = 0.002) and cervical cancers (*P*<0.001) (**Fig 1B**).

**Table 1:** Association of TPP1 expression and clinicopathological characteristics of cervical cancer.

	TPP1 expression		
Characteristics	High	Low	*P* value
**Age (years)**			0.643
≤45	71	19	
>45	50	16	
**HPV infection**			0.689
Negative	17	4	
Positive	104	31	
**Tumor diameter (cm)**			0.376
≤4	73	24	
>4	48	11	
**FIGO stage**			0.990
I	52	15	
II	69	20	
**Differentiation**			0.006
Poorly	60	8	
Moderately	51	19	
Well	10	8	
**Lymphatic invasion**			0.047
Negative	71	27	
Positive	50	8	
**Vagina invasion**			0.022
Invasion	43	20	
No invasion	78	15	
**Parauterus invasion**			0.480
Invasion	34	12	
No invasion	87	23	
**Pathologic type**			0.847
SCC	107	32	
AC/ASC	14	3	

Abbreviations: AC: Adenocarcinoma; ASC: Adenosquamous carcinoma; FIGO: The International Federation of Gynecology and Obstetrics; HPV: Human papillomavirus; SCC: Squamous cell carcinoma; TPP1: Telomere-binding protein TINT1-PTOP-PIP1.

In agreement with increased proportion of high TPP1 expression detected in CINs and cervical cancer samples, higher expression of the *TPP1* gene was validated in external and publicly available databases (GSE7803 and Oncomine). In the GSE7803 dataset, 10 normal cervix, 7 CINs, and 21 cervical cancers were identified. When compared with normal tissues, *TPP1* was higher expressed in CINs (*P*<0.05) and strongly expressed in cervical carcinoma (*P*<0.001) (**Fig 1C**). Similarly, in the Zhai Cervix dataset from Oncomine that included 10 normal cervical tissues and 21 cervical SCC cases, the *TPP1* gene was significantly highly expressed in cervical SCC compared with normal cervical tissues (*P*< 0.001) (**Fig 1D**).

For the clinical-pathological factors of cervical cancer, TPP1 expression was related to pathological differentiation (*P* = 0.006), lymphatic invasion (*P* = 0.047), and vaginal invasion (*P* = 0.022) **(Table 1)**.

### Correlation of TPP1 and hTERT expression

Expression of TPP1 and hTERT were measured in the same batch of samples and compared in different cervical lesions (n = 274). Similar to TPP1, the percentage of patients with high expression of hTERT increased in CIN3 and cervical cancers (**Table 2, S1A Fig**). A strong agreement between TPP1 expression and hTERT expression was also found in CIN 3 (Kappa statistics = 0.50, *P* = 0.005), SCC (Kappa statistics = 0.22, *P* = 0.011), and AC/ASC patients (Kappa statistics = 0.77, *P* = 0.001) (**Table 2**).

**Table 2:** Correlation of expression of TPP1 and hTERT in cervical tissues.

Tissue type	TPP1 high, n	TPP1 low, n	Concordance (%)	Kappa statistics (95%CI)	*P* value
**Normal tissue**					
hTERT high	1	3	81.3	0.33 (-0.17–0.84)	0.074
hTERT low	0	12			
**CIN 1**					
hTERT high	3	25	48.1	0.02 (-0.13–0.17)	0.772
hTERT low	2	22			
**CIN 2**					
hTERT high	4	4	68.2	0.29 (-0.12–0.71)	0.166
hTERT low	3	11			
**CIN 3**					
hTERT high	13	1	75.0	0.50 (0.20–0.80)	0.005
hTERT low	6	8			
**SCC**					
hTERT high	87	19	71.9	0.22 (0.04–0.40)	0.011
hTERT low	20	13			
**AC/ASC**					
hTERT high	14	1	94.1	0.77 (0.34–1.00)	0.001
hTERT low	0	2			
**All**					
hTERT high	128	57	70.1	0.38 (0.27–0.48)	<0.001
hTERT low	25	64			

Abbreviations: AC: Adenocarcinoma; ASC: Adenosquamous carcinoma; CI: Confidence interval; CIN: Intraepithelial neoplasia; hTERT: Human telomerase reverse transcriptase; SCC: Squamous cell carcinoma; TPP1: Telomere-binding protein TINT1-PTOP-PIP1.

**Fig 2:**
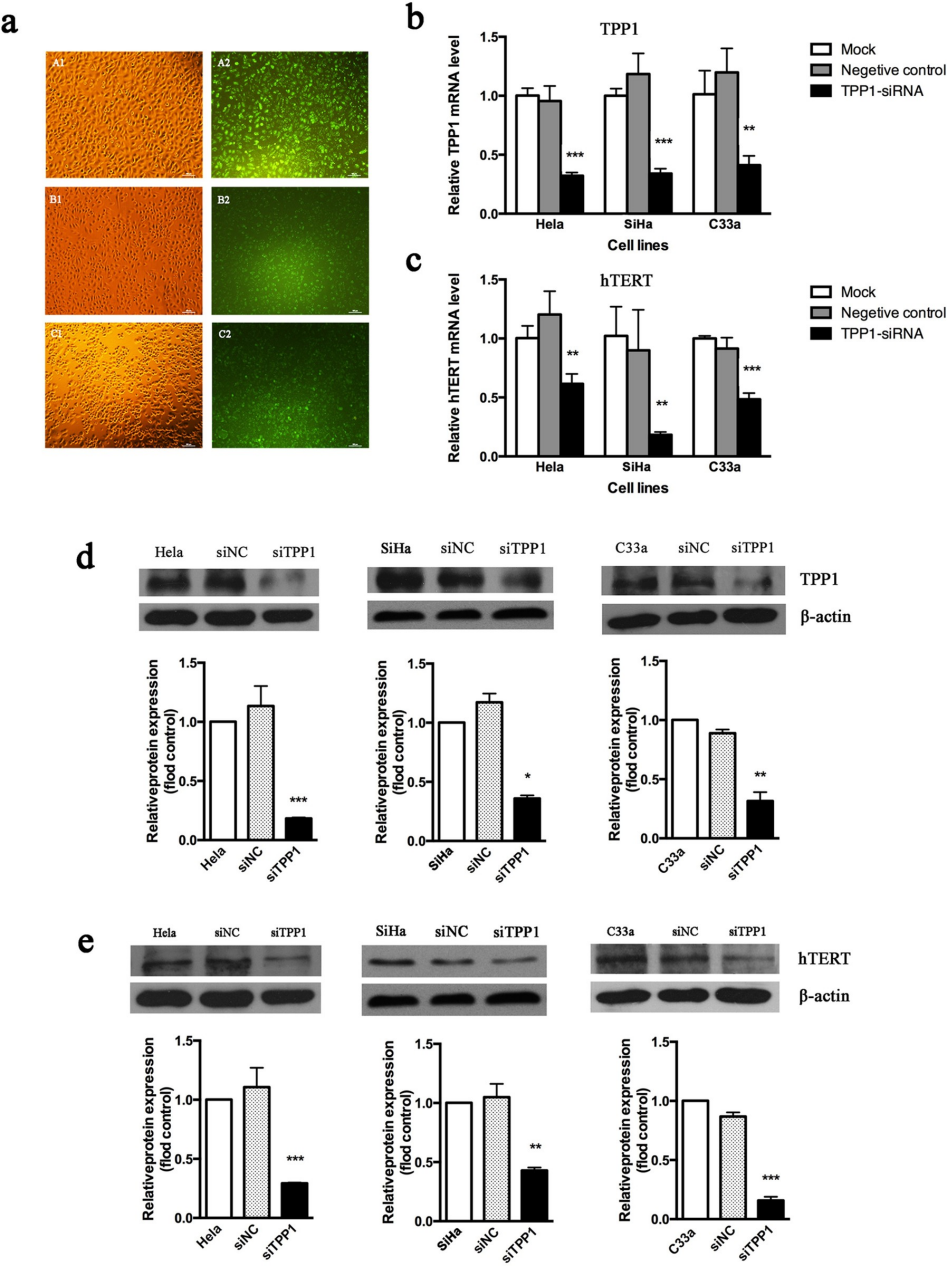
Knockdown of TPP1 influenced the expression of hTERT in three cervical cancer cell lines. **(a)** Representative fluorescence images verifying the knockdown effectiveness in HeLa (A1, A2), SiHa (B1, B2), and C33a (C1, C2) cervical cancer cell lines after transfection with siTPP1 (100×, scale: 100 μm). The left panel showed bright field images of three cell lines, and the right panel with green fluorescence indicated successful transfection by the plasmids of the corresponding cell lines. **(b)** *TPP1* mRNA expression was decreased, as detected by real-time qPCR, after siTPP1 transfection. **(c)** *hTERT* mRNA expression was decreased after TPP1 knockdown, as detected by real-time qPCR. **(d)** Decreased TPP1 protein expression detected by western blot after siTPP1 transfection. **(e)** hTERT protein expression assessed by western blot was decreased after *TPP1* knockdown. Abbreviations: NC: Negative control; * *P*<0.05, ** *P*<0.01, *** *P*<0.001, n = 3.

To explore the regulatory effect of TPP1 on hTERT, we transfected the *TPP1*-siRNA into three human cervical cancer cell lines. Fluorescence microscopy, real-time qPCR, and western blotting were used to confirm the transfection efficiency and depletion of TPP1. The transfection efficiencies in HeLa, SiHa, and C33a cells were 95%, 90%, and 85%, respectively (**Fig 2A**). Compared with control cells, mRNA levels of *TPP1* and *hTERT* in three cell lines were significantly reduced in the *TPP1*-siRNA transfected cells (**Fig 2B and 2C**). We also found the protein expression of both TPP1 and hTERT were downregulated in *TPP1*-depleted cells in all these cell lines (**Fig 2D and 2E**). Compared with their mock controls, *hTERT* mRNA expression in HeLa, SiHa, and C33a cells was reduced by 38.5%, 81.7%, and 51.5%, respectively (**Fig 2C**). The deduced expression levels of the hTERT protein were 70.7%, 57.0%, and 85.2%, respectively (**Fig 2E**). These results suggested that TPP1 possibly influenced hTERT expression in cervical cancer cells.

### TPP1 expression and overall survival in cervical cancer

**Fig 3:**
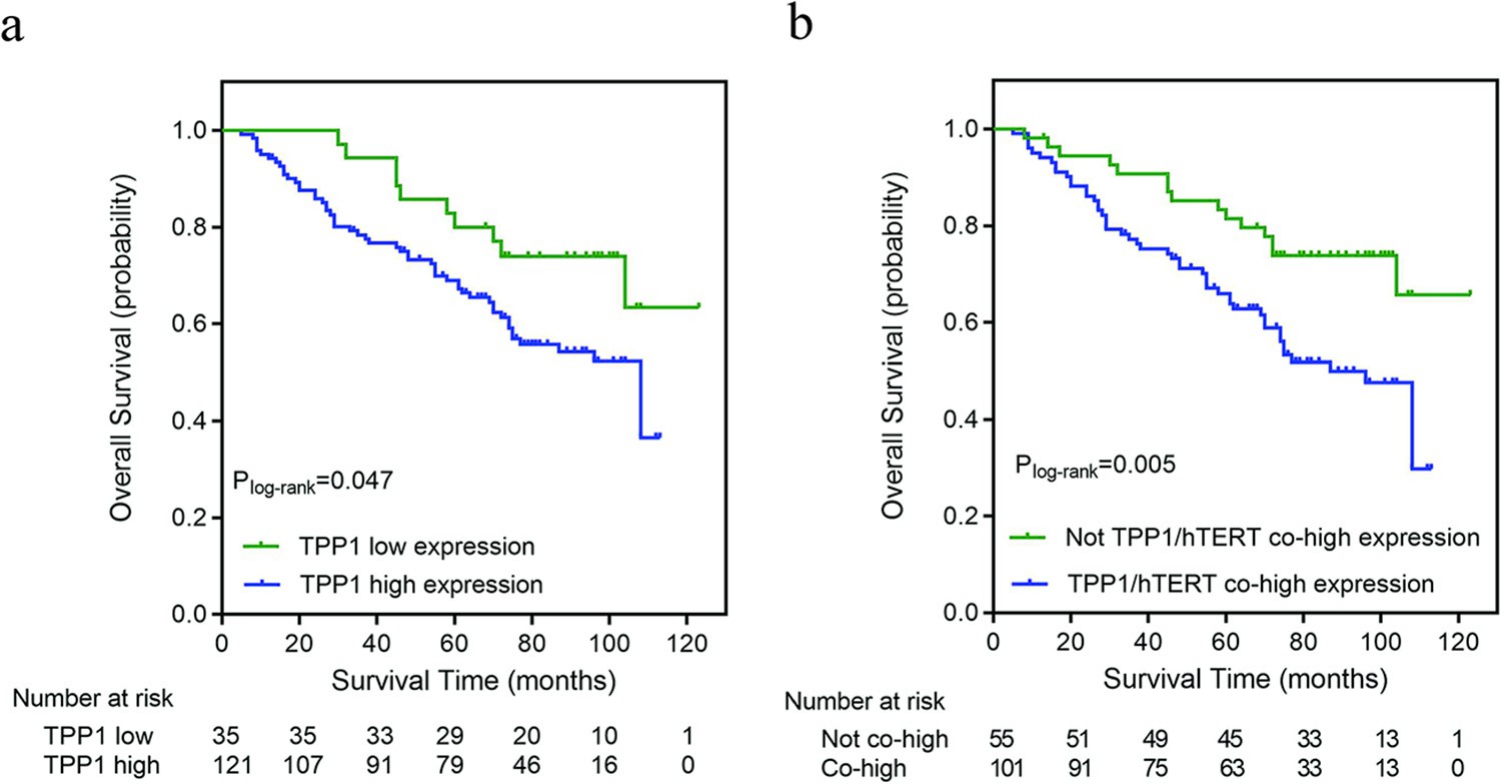
Kaplan–Meier curve of overall survival among cervical cancer patients by expression levels of TPP1 or together with hTERT. **(a)** High expression of TPP1 was associated with worse overall survival in cervical cancer patients (n = 156). **(b)** Patients with high expression of both TPP1 and hTERT had worse overall survival than those without co-high expression of TPP1 and hTERT (n = 156).

The median follow-up time for cervical cancer patients was 93 months. Among 156 cervical cancer patients, 65 (41.7%) died during the follow-up, generating a median overall survival of 108 months. Compared with cervical cancer patients with low TPP1 expression, significantly worse survival was found in those with high expression of TPP1 (*P*_log-rank_ = 0.047, **Fig 3A**).

Multivariable analysis demonstrated that high expression of TPP1 was independently associated with poorer survival in cervical cancer (HR = 2.61 [95% CI 1.23–5.51], *P* = 0.012, **Table 3**). Kaplan–Meier analysis showed that higher expression of hTERT tended to be associated with worse survival, although the difference was not statistically significant (*P*_log-rank_ = 0.060, **S1B Fig**). A total of 64.7% (101/156) of cancer patients had co-high expression of both TPP1 and hTERT (**Table 3**). Patients with co-high expression of TPP1/hTERT had significantly worse overall survival (*P*_log-rank_ = 0.005, **Fig 3B**), carrying a 2.38 (95% CI 1.28–4.43, *P* = 0.006) times higher mortality risk than those without co-high expression (**Table 2**).

**Table 3:** Multivariable-adjusted hazard ratio of overall survival among cervical cancer patients.

	No. death	No. patients	Cox regression model 1^a^		Cox regression model 2 ^b^	
			Hazard ratio (95% CI)	*P* value	Hazard ratio (95% CI)	*P* value
**Age (years)**	65	156	1.04 (1.01–1.08)	0.006	1.04 (1.01–1.07)	0.014
**HPV infection**						
Negative	13	21	Reference	0.235	0.67 (0.34–1.34)	0.261
Positive	52	135	0.66 (0.33–1.31)			
**Tumor diameter (cm)**	65	156	1.02 (0.83–1.25)	0.872	1.02 (0.84–1.25)	0.826
**FIGO stage**						
I	17	67	Reference	0.002	Reference	0.002
II	48	89	3.40 (1.59–7.27)		3.37 (1.56–7.28)	
**Differentiation**						
Poor	30	68	Reference	0.191	Reference	0.188
Moderate	26	70	0.90 (0.49–1.64)		0.79 (0.44–1.43)	
Well	9	18	2.02 (0.85–4.79)		1.80 (0.77–4.20)	
**Lymphatic invasion**						
Negative	35	98	Reference	0.411	Reference	0.420
Positive	30	58	1.27 (0.72–2.26)		1.26 (0.72–2.23)	
**Vagina invasion**						
Negative	33	93	Reference	0.903	Reference	0.953
Positive	32	63	1.04 (0.55–1.96)		0.98 (0.52–1.84)	
**Parauterus invasion**						
Negative	42	110	Reference	0.829	Reference	0.874
Positive	23	46	1.07 (0.60–1.90)		1.05 (0.59–1.88)	
**Pathological type**						
SCC	57	139	Reference	0.678	Reference	0.550
AC/ASC	8	17	0.84 (0.36–1.94)		0.77 (0.33–1.79)	
**Chemoradiotherapy**						
No chemo- nor radiotherapy	13	38	Reference	0.051	Reference	0.060
Chemotherapy only	8	23	0.47 (0.17–1.34)		0.42 (0.15–1.22)	
Radiotherapy only	11	32	0.62 (0.25–1.51)		0.60 (0.24–1.48)	
Both chemo- and radiotherapy	30	59	1.27 (0.58–2.81)		1.21 (0.54–2.68)	
Unknown	3	4	3.02 (0.76–11.97)		2.42 (0.62–9.46)	
**TPP1 expression**						
Low expression	10	35	Reference	0.012	-	-
High expression	55	121	2.61 (1.23–5.51)		-	
**TPP1/hTERT co-high expression**						
Without co-high expression	15	55	-	-	Reference	0.006
With co-high expression	50	101	-		2.38 (1.28–4.43)	

Abbreviations: AC: Adenocarcinoma; ASC: Adenosquamous carcinoma; CI: Confidence interval; FIGO: The International Federation of Gynecology and Obstetrics; hTERT: Human telomerase reverse transcriptase; SCC: Squamous cell carcinoma; TPP1: Telomere-binding protein TINT1-PTOP-PIP1.

^a^ Cox regression model 1 included all covariates listed in the table except for TPP1/hTERT co-high expression.

^b^ Cox regression model 2 included all covariates listed in the table except for TPP1 expression.

## Discussion

This study indicated that high TPP1 expression was significantly related to CIN 3 and cervical cancers but not CIN 1 or CIN 2. TPP1 expression positively correlated with the expression of hTERT in cervical cancer, and depletion of *TPP1* substantially decreased hTERT expression. In addition, high TPP1 expression and co-highly expressed with hTERT were both independent predictors of poor survival for cervical cancer patients. These findings indicated that TPP1 might be a crucial pathogenesis and prognosis factor of cervical cancer, advancing cancer malignancy partly by influencing hTERT expression.

The mechanisms of TPP1 in determining cervical cancer development remain unclear. Previous studies found that higher expression of TPP1 was involved in telomere elongation and contributed to the increase in malignant potential in B cell leukemia and colorectal cancer [18,19]. TPP1 was overexpressed in gastric cancer compared to adjacent normal tissue and confirmed that TPP1 promoted cancer cell proliferation [20]. The present study indicated that TPP1 was related to the progression of cervical cancer malignancy, as shown by the constant increase of high TPP1 expression rates from the normal cervix, CIN 1, CIN 2, CIN 3 to SCC and AC/ASC. Ki-67 encoded by the *MKI67* gene is a marker of cellular proliferation and is closely related to tumor malignancy. As an exploration, we also assessed the correlation between *TPP1* and *MKI67* gene expression using TCGA data from the GEPIA database in cervical cancer. Interestingly, we found that mRNA expression of *TPP1* was positively correlated with the expression of *MKI67* (S2 Fig), suggesting that *TPP1* could be linked to the proliferation and malignancy of cervical cancer. Additionally, our results suggested that increased TPP1 expression was an early event during epithelial malignant occurrence, and a substantial-high expression rate already occurred in CIN 3. The result was verified by a small external analysis when we analyzed the expression of *TPP1* in the GSE7803 dataset. Our study suggested that TPP1 might be a potential predictive biomarker for CIN 3 and cervical cancers.

TPP1 is responsible for recruiting telomerase to the telomere and maintaining telomerase processivity with the inseparable participation of hTERT [21]. Correlative analysis of clinical samples in the present study revealed that TPP1 and hTERT expression levels were positively associated. TPP1 is crucial for hTERT recruitment to telomeres and telomere elongation [22]. The *in vitro* results verified that the expression of hTERT was decreased at both mRNA and protein levels when TPP1 expression was knocked down. Although direct proof of hTERT regulation by TPP1 is currently lacking, we speculated that hTERT might be regulated by TPP1 through several pathways. One possible way could be that the TEL patch on the surface of TPP1 directly interacted with the TEN domains of hTERT to recruit telomerase to telomeres [23,24]. Knockdown of TPP1 decreased telomerase activity by affecting the TPP1 and hTERT combination, which further reduced hTERT expression. Additionally, some studies reported that TPP1 bound to POT1 and interacted with POT1 to affect telomerase regulation at chromosome ends [25]. The C-terminal portion of human POT1 (POT1C) is vital for the POT1C-TPP1 interaction, and disruption of the interaction is essential to prevent the initiation of genome instability permissive for tumorigenesis [26]. hTERT activity could be partially activated by overexpression of TPP1-POT1 via the insertion of the finger domain (IFD) at hTERT in a TPP1-dependent manner [27]. Both of these pathways are critical for the functions of TPP1-hTERT [21]. Moreover, c-Myc is involved in regulating the TPP1-hTERT complex via phosphatidylinositol 3-kinase/Akt, which regulates vascular smooth muscle cell cycle progression and cell proliferation [28]. In addition, other research found that TPP1 might also be involved in the regulation of telomere shortening by being an upstream regulator of hTERT in human cells [29]. It is possible that TPP1 expression is elevated earlier than the activation of hTERT and that hTERT activation by TPP1 signaling pathway might lead to the occurrence or progression of cervical cancer.

It was interesting to note that high TPP1 expression was an independent predictor of poor survival for cervical cancer and related to tumor differentiation, lymphatic invasion, and vagina invasion, indicators of aggressiveness of this disease. High expression of TPP1 in tumor cells can protect the telomere end structure from fusion and DNA damage and thus enhance genomic stability and improve cellular immortalization potential [30]. Telomerase of tumor cells might be highly activated when both TPP1 and hTERT were overexpressed, which could further result in higher invasiveness and poorer prognosis.

We also performed an exploratory analysis of the expression difference of the *TPP1* gene in other cancer types and their corresponding controls using the Oncomine database. The results revealed that *TPP1* was also overexpressed in several other cancers, such as esophageal carcinoma, stomach adenocarcinoma, and liver hepatocellular carcinoma, and increased expression of TPP1 was associated with worse survival in these cancers (S3 Fig). Overexpressed TPP1 might promote tumor malignancy in specific cancers, implying the possibility of TPP1 being a prognostic predictor of these cancers with high TPP1 expression.

The strengths of the study include rigorous clinical data collection, a long-term follow-up, an exploration of potential molecular mechanisms concerning TPP1 and hTERT, and extended bioinformatics analysis. *In vitro* experiments using three different well-known cervical cancer cell lines were well designed, and each finding was validated through several methods, ensuring the robustness of the results. However, several limitations warrant attention. Although we enrolled all cervical cancer patients during the study period, this was a single hospital-based study with a limited study sample size, which could hinder its generalizability. Therefore, further large prospective studies in different populations are warranted to evaluate the clinical application of TPP1. This study is also limited by lacking non-cancerous cell lines as controls due to the availability of such tool. The mechanism of how TPP1 interacts with hTERT and how TPP1 promotes the malignant phenotype of cervical cancer requires further investigation.

In conclusion, TPP1 expression increased significantly in late precursor lesions (CIN 3) and cervical cancers compared and high expression of TPP1 indicated worse survival in cervical cancer. TPP1 and hTERT expression were positively correlated by both *in vivo* and *in vitro* analysis. This study indicate high expression of TPP1 may be an early event during cervical cancer development and a prognostic factor for survival in cervical cancer.

## Supporting information

S1 Fig Expression of hTERT in different cervical lesions and association with overall survival among cervical cancer patients. **(a)** hTERT expressions in normal, CIN 1, CIN 3 and cervical carcinoma tissues by immunohistochemistry (n = 274, scale 100μm). **(b)** High expression of hTERT was marginally associated with worse overall survival in cervical cancer patients (n = 156). Abbreviations: AC: Adenocarcinoma; ASC: Adenosquamous carcinoma; CINs: Intraepithelial neoplasia; SCC: Squamous cell carcinoma; * *P*<0.05, *** *P*<0.001.
(TIFF)

S2 Fig Significant correlation between *TPP1* and *MKI67* gene expressions in cervical cancer, evaluated by TCGA data from GEPIA database. (TIF)

S3 Fig Examples of TPP1 expression in other cancers and association of its high expression and cancer survival. **(a)** Expression of TPP1 in cancer of esophagus, stomach, and liver compared with normal tissues from TCGA and GTEx databases using GEPIA. **(b)** The Kaplan-Meier curve of TPP1 expression in normal tissues, and cancer of esophagus, stomach, and liver. * *P*<0.05.
(TIF)

S1 Table Scoring system for hTERT positive cells according to the intensity and distribution. (PDF)

S2 Table Primers designed for GAPDH, hTERT, and TPP1. (PDF)

S1 Raw images (PDF)

S1 Data (XLSX)
