# Supplementary figures and images for: TPP1 is associated with risk of advanced precursors and cervical cancer survival
Source: PLoS One. 2024 May 9;19(5):e0298118. doi: 10.1371/journal.pone.0298118 (PMC11081309; doi:10.1371/journal.pone.0298118)

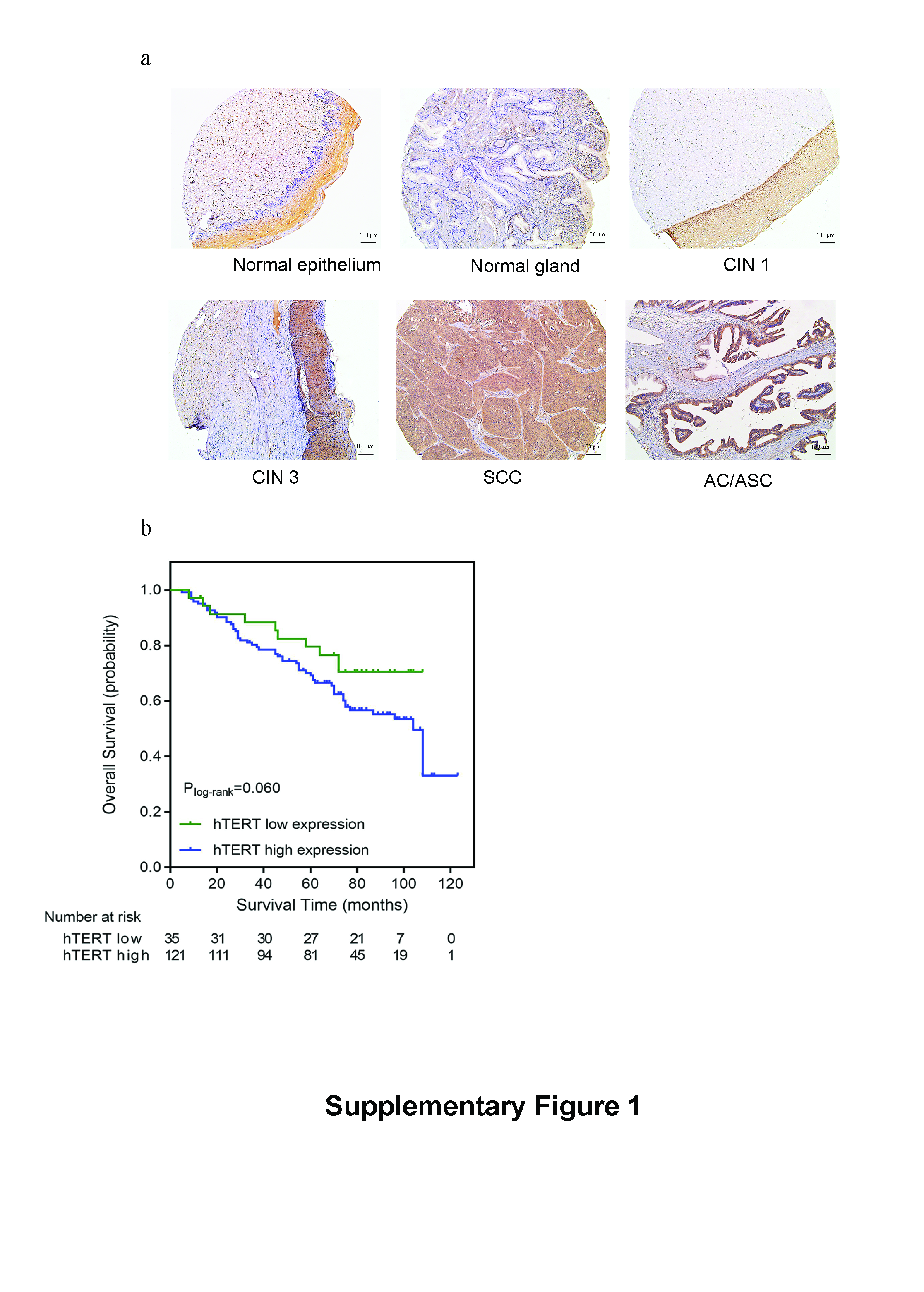

Supplement: S1 Fig — (a) hTERT expressions in normal, CIN 1, CIN 3 and cervical carcinoma tissues by immunohistochemistry (n = 274, scale 100μm). (b) High expression of hTERT was marginally associated with worse overall survival in cervical cancer patients (n = 156). Abbreviations: AC: Adenocarcinoma; ASC: Adenosquamous carcinoma; CINs: Intraepithelial neoplasia; SCC: Squamous cell carcinoma; * P<0.05, *** P<0.001. (TIFF) [file pone.0298118.s001.tiff]

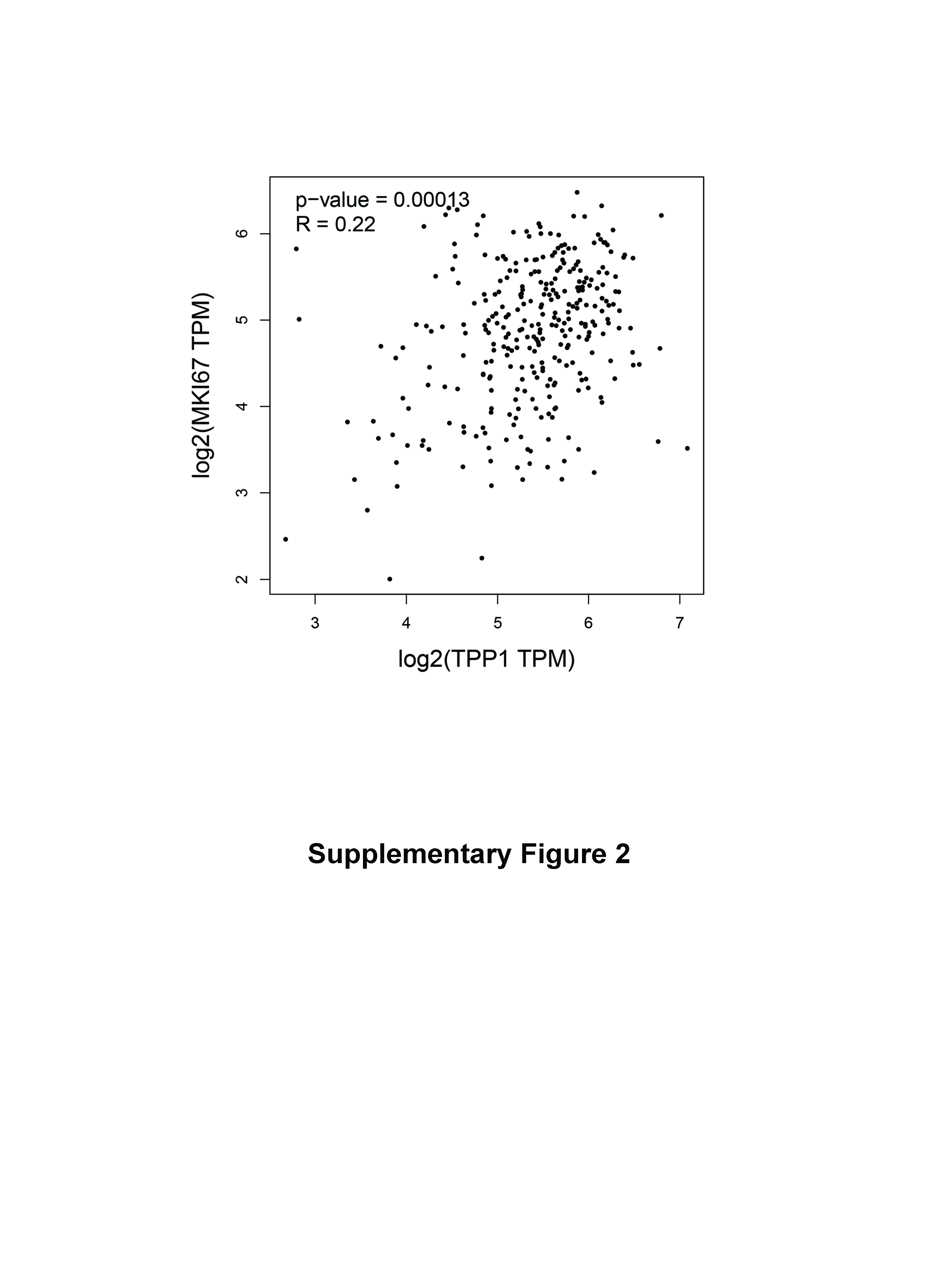

Supplement: S2 Fig — (TIF) [file pone.0298118.s002.tif]

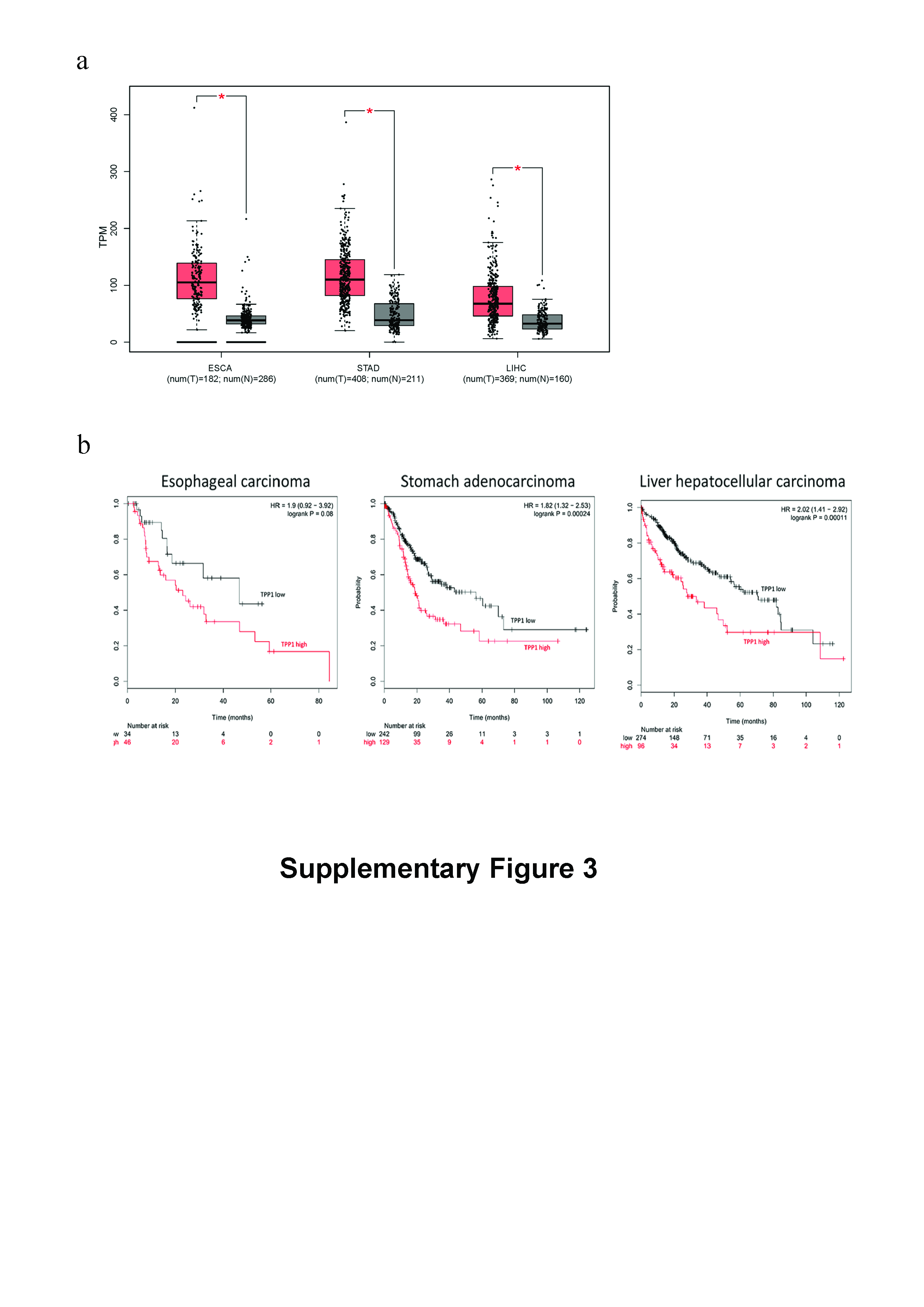

Supplement: S3 Fig — (a) Expression of TPP1 in cancer of esophagus, stomach, and liver compared with normal tissues from TCGA and GTEx databases using GEPIA. (b) The Kaplan-Meier curve of TPP1 expression in normal tissues, and cancer of esophagus, stomach, and liver. * P<0.05. (TIF) [file pone.0298118.s003.tif]
