# Supplementary material for: TPP1 is associated with risk of advanced precursors and cervical cancer survival
Source: PLoS One. 2024 May 9;19(5):e0298118. doi: 10.1371/journal.pone.0298118 (PMC11081309; doi:10.1371/journal.pone.0298118)
Supplement: S1 Table — (PDF) [file pone.0298118.s004.pdf]

S1 Table. Scoring system for hTERT positive cells according to the intensity and distribution.

Protein	Parameters		Score
**TPP1**			
	Positive cells (%)		
		<25%	0
		25-49%	1
		50-74%	2
		>75%	3
	Overall score (positive cells)		
		Low expression	0-1
		High expression	2-3
**hTERT**			
	Positive cells (%)		
		<10%	0
		10-50%	1
		50-75%	2
		>75%	3
	Intensity of staining		
		Negative (no staining)	0
		Weak (yellow)	1
		Moderate (brown)	2
		Strong (dark brown)	3
	Overall score (positive cells × intensity) *		
		Low expression	0-3
		High expression	4-9

*An overall score was obtained by multiplying the intensity score and the positive score.
