# Supplementary material for: TPP1 is associated with risk of advanced precursors and cervical cancer survival
Source: PLoS One. 2024 May 9;19(5):e0298118. doi: 10.1371/journal.pone.0298118 (PMC11081309; doi:10.1371/journal.pone.0298118)
Supplement: S2 Table — (PDF) [file pone.0298118.s005.pdf]

S2 Table. Primers designed for GAPDH, hTERT, and TPP1

Primer	Sequence
**GAPDH**	
forward primer	5’-TGGAAGGACTCATGACCACA-3’
reverse primer	5’-TTCAGCTCAGGGATGACCTT-3’
**hTERT**	
forward primer	5’-CGGAAGAGTGTCTGGAGCAA-3’
reverse primer	5’-GGATGAAGCGGAGTCTGGA-3’
**TPP1**	
forward primer	5’-ATCTCGAAGTATGCCTGGCCGCTGTCAGAGTG-3’
reverse primer	5’-AGCGGCCGCTATCACATCGGAGTTGGCTCAGAC-3’
