# Supplementary material for: TPP1 is associated with risk of advanced precursors and cervical cancer survival
Source: PLoS One. 2024 May 9;19(5):e0298118. doi: 10.1371/journal.pone.0298118 (PMC11081309; doi:10.1371/journal.pone.0298118)
Supplement: S1 Raw images — (PDF) [file pone.0298118.s006.pdf]

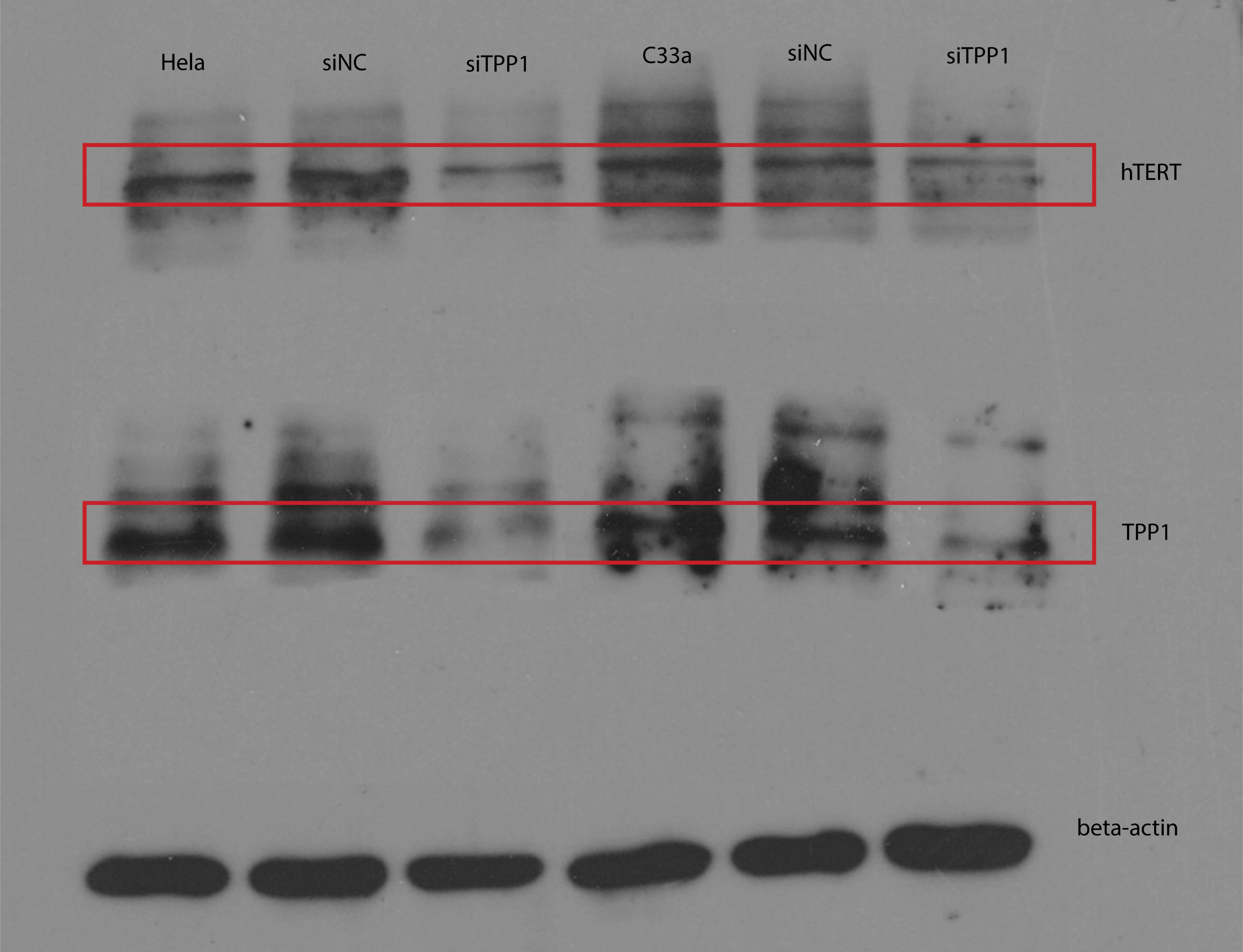

Figure 2d & 2e. hTERT and TPP1 expression of Hela and C33a cells after TPP1 knockdown

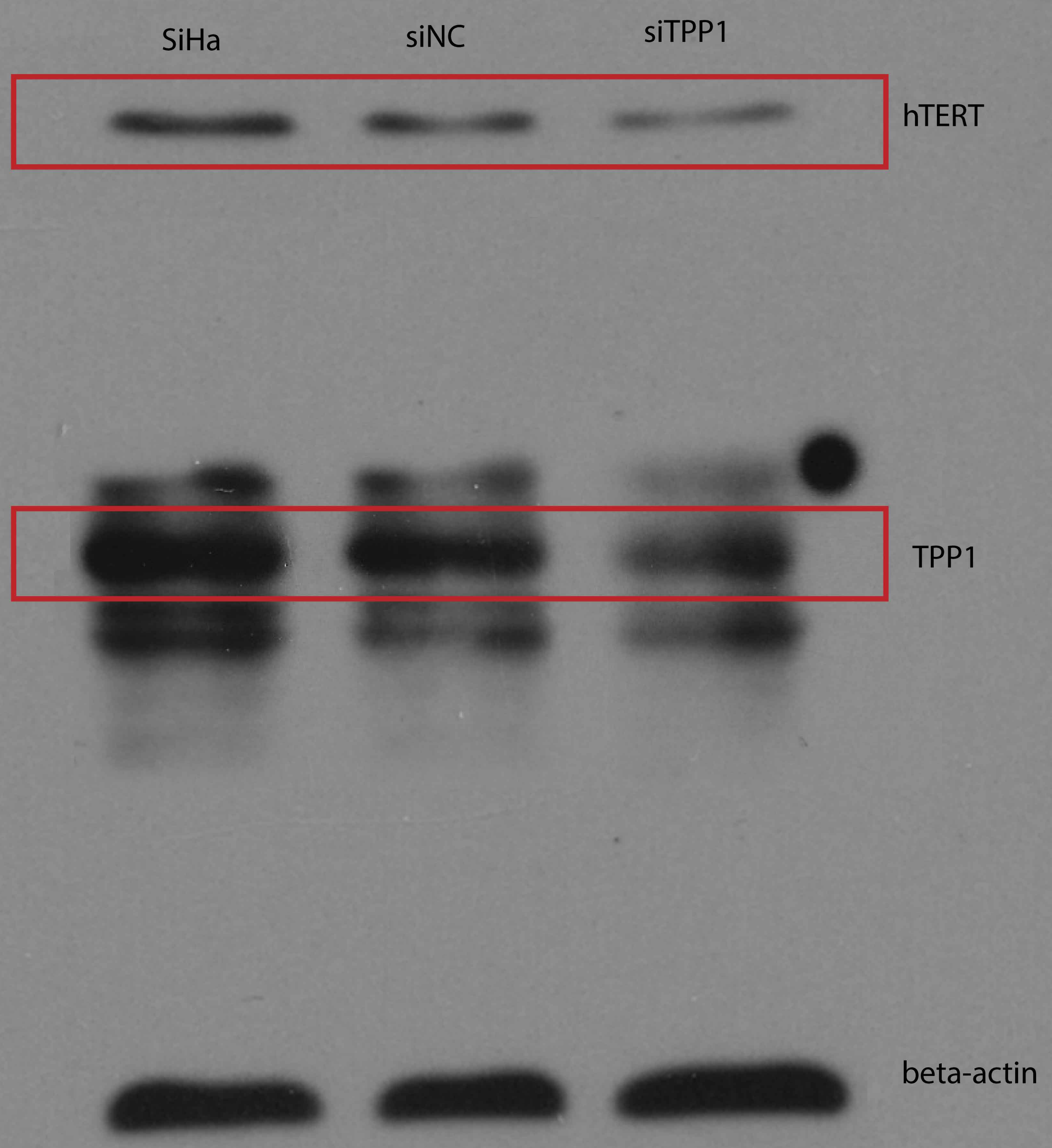

Figure 2d & 2e. hTERT and TPP1 expression of SiHa cells after TPP1 knockdown
